# A double-edged sword: The effects of R&D intensity and capitalization on institutional investment in entrepreneurial firms

**DOI:** 10.3389/fpsyg.2022.942931

**Published:** 2022-09-23

**Authors:** Yuan Feng, Chenyang Ma, Yushi Wang, Jiangshui Ma

**Affiliations:** ^1^School of Accounting, Southwestern University of Finance and Economics, Chengdu, Sichuan, China; ^2^School of Business Administration, Southwestern University of Finance and Economics, Chengdu, Sichuan, China

**Keywords:** R&D intensity, R&D capitalization, institutional investment, entrepreneurial firms, venture capital

## Abstract

Studies show that research and development (R&D) may not always benefit entrepreneurial firms. This paper focuses on the double-edged effect of R&D activities on attracting institutional investment in entrepreneurial firms. Based on a panel dataset of 700 listed entrepreneurial firms in ChiNext, we document: (1) an inverted-U relationship between R&D intensity and future institutional investment, which we argue is evidence that institutional investors are concerned about R&D overinvestment; (2) an inverted-U relationship between R&D capitalization and future institutional investment, which we argue shows suspicion of the institutional investors towards high R&D capitalization. Furthermore, by splitting institutional investors into venture capitals (VCs) and non-venture capitals (non-VCs), we confirm that VCs have higher acceptance of both R&D intensity and capitalization as VCs have more expertise to alleviate a certain level of risks.

## Introduction

Deciding how much resources to allocate to R&D activities is critical for entrepreneurial firms’ survival and growth. R&D spending can have a double-edged effect on start-up firms: if the spending is insufficient, potential competitive advantage can be lost; and if the spending is too large, R&D activities become a financial burden, exhausting resources and speeding up failures (Chen, 2008; Artz et al., 2010; Delmar et al., 2013; Ugur et al., 2016; Grimpe et al., 2017). Therefore, an *optimum* level of R&D investments should exist for entrepreneurial firms theoretically, while studies have found multiple factors that deviate actual R&D investments from the *optimum* level. These factors include agency conflicts, ownership, the social and legal environment for R&D underinvestment, characteristics of R&D investments, and the competitive environment for R&D overinvestment (Osma and Young, 2009; Denicolò and Zanchettin, 2014; Gomez-Mejia et al., 2014; Ahuja and Novelli, 2017; Xu and Yano, 2017). Despite a growing literature studying factors deviating R&D investments from the *optimum* level, there is little research on how the capital market responds to such deviation.

In this paper, based on these prior findings, we study how institutional investors, the sophisticated group of the capital market, respond to R&D investments. Because of the double-edged feature of R&D investments on shareholders’ value, we hypothesize that an *optimum* level of R&D intensity exists concerning institutional investment. Moreover, as a critical aspect of R&D investments, R&D capitalization represents R&D success but can also be a way for managers to manipulate earnings, so we also study whether a *credible* limit of R&D capitalization exists in attracting institutional investments.

To test our hypothesis, we examine the double-edged sword effect of R&D intensity and capitalization by examining a dataset from 700 listed entrepreneurial firms in ChiNext. We find an inverted-U relationship between R&D intensity and future institutional investment, suggesting that institutional investors worry about R&D overinvestment above the *optimum* level. Also, our results confirm an inverted-U relationship between R&D capitalization and future institutional investment, indicating that institutional investors are concerned about *suspicious* R&D capitalization past the *credible* limit.

To further study the research question, we split institutional investors into venture capitals (VCs) and non-venture capitals (non-VCs). Because VCs are usually more long-term oriented and specialized, we argue that they can distinguish the overinvestment and *suspicious* capitalization to have a higher *optimum* level and *credible* limit. We find evidence that an *optimum* level of R&D intensity and a *credible* limit of R&D capitalization exist only for non-VCs in our sample, while VCs have a linearly positive relationship with both the intensity and capitalization.

We make three main contributions. First, extant literature has paid little attention to the R&D overinvestment and its effect on entrepreneurial firms; we uncover that R&D overinvestment discourages future institutional investment. Second, in line with previous studies (Dinh et al., 2016; Kreß et al., 2019), we show that institutional investors consider the credibility of R&D capitalization. Third, by examining the effects on VCs and non-VCs, we confirm that VCs are less concerned about R&D overinvestment and *suspicious* capitalization as they can make a distinction.

## Theory framework

A conventional view of R&D activities is that firms follow a rule of thumb that seeks the same R&D intensity as competitors in the same industry (Grabowski and Baxter, 1973). Thus, the level of R&D intensity should be homogenous in the same industry (Cincera and Veugelers, 2013). However, many empirical studies have documented that this assumption does not hold. For example, heterogeneity in R&D intensities exists across industries and within the same industry (Leiponen and Drejer, 2007; Coad, 2019). Next, we will explain the heterogeneity in R&D intensities from the view of under-and over-investment.

### Under- and over-investment in R&D

The prevailing view of R&D efforts is that firms tend to underinvest in R&D. R&D underinvestment is defined as the private firm level of R&D investment falling well below the socially optimal level (Jones and Williams, 2000; Brown et al., 2017). There are several reasons for the underinvestment. First, firms cut R&D spending in response to short-term earnings pressure. From an agency theory perspective, corporate managers act out of self-interest, using reductions in R&D intensity to meet short-term earnings targets (Osma and Young, 2009; Xu et al., 2022). The agency problem is also enhanced by analyst coverage and forecasts, especially when managers face an increase in employment risk after missing the forecasts (Gentry and Shen, 2013). Likewise, the myopic R&D investment decision is encouraged by high turnover and momentum trading by institutional investors (Bushee, 1998).

Second, R&D intensities are affected by corporate governance practices, and family ownership may discourage risky long-term R&D investment (Chen and Hsu, 2009; Block, 2012). Gomez-Mejia et al., 2014 explain that family underinvestment in R&D is caused by the families’ balancing of socioemotional gains and losses using a behavioral agency model. Further, in terms of transgenerational leadership transitions, the successors will focus on short-term developments and invest less in R&D activities due to the close watch from outgoing leaders and company stakeholders (Li et al., 2022). In addition, conflicts and an unhealthy dialogue between top management teams and boards can compromise the R&D investment (Kor, 2006).

Third, firms’ R&D investments are also affected by the legal and social environment. In countries where legal protections for intellectual property are weak, firms underinvest in R&D due to an insufficient private return (Brown et al., 2017). Because of bureaucrats’ rent-seeking behavior, corruption also discourages firms from investing in R&D (Xu and Yano, 2017).

All combined, extant studies have made a considerable effort to investigate R&D underinvestment, while the overinvestment in R&D is less identified in prior studies. Specific characteristics of R&D investments, such as uncertainty, boundary ambiguity, feedback latency, R&D lumpiness, and legitimacy, make overinvestment likely to occur (Ahuja and Novelli, 2017).

From a view of the competitive economy, two distortions account for the overinvestment in R&D. Jones and Williams (2000) show that out of patent races, firms are promoted to overinvest R&D by the stepping-on-toes effect (i.e., an increase in R&D decreases the probability of competitors’ success) and the redistribution of rents effect (i.e., the new innovator takes all profits and past innovator gets cut out). Denicolò and Zanchettin (2014) provide further analysis supporting the stepping-on-toes effect.

While economists look at the R&D overinvestment issue from a social welfare perspective, other researchers focus more on the individual firm’s perspective. Aoki (1991) concludes that firms have to be persistently highly R&D active because their survival depends on successful products or the R&D technology and knowledge; however, the substantial consumption of financial resources on R&D may threaten entrepreneur firms’ survival (Delmar et al., 2013). R&D activities may be a passive response to industry competition (Lee, 2009; Thakor and Lo, 2021). In summary, instead of guaranteeing future success, R&D activities may be a waste and an overinvestment from an individual firm’s perspective.

### R&D capitalization

Managers use their discretion over whether to capitalize or to expense R&D costs. R&D capitalization means sustaining R&D costs in the balance sheets as new assets, signaling further revenue, rather than expensing them in the income statements, lowering current profits. While the US generally accepted accounting principles (GAAP) mandates immediate expensing of all R&D costs, international financial reporting standards (IFRS) and Chinese Accounting Standards (CAS) prescribe capitalization of development costs when they meet all criteria.^1^ The criteria evaluate the likelihood that future economic benefits will flow into the firms by testing for both the technical and commercial feasibility; however, this conditional capitalization policy leaves firms flexibility in deciding whether to recognize the cost as an asset because the criteria are not explicit enough and no further guidance is available to determine the threshold of the feasibility (Jones, 2011).

As a result, R&D capitalization can be either *credible* or *suspicious*. R&D capitalization can signal success in R&D projects and potential future benefits for investors (Lev and Zarowin, 1999; Oswald, 2008). However, Dinh et al. (2016) point out that only the R&D capitalization truthfully reflecting R&D success, which we refer to as *credible* R&D capitalization, is positively valued. *Suspicious* R&D capitalization happens when managers manipulate earnings or signal fake information. On the one hand, managers may untruthfully capitalize more R&D costs to smoothen earnings (Markarian et al., 2008); beat earnings targets (Cazavan-Jeny et al., 2011; Dinh et al., 2016); or respond to financial distress (Jones, 2011). On the other hand, Kreß et al. (2019) show that capitalized R&D significantly rose before debt financing. Together, driven by these motives to manipulate earnings and convey falsely optimistic signals, managers tend to capitalize on higher-than-normal R&D costs.

### R&D activities in entrepreneurial firms

Research and development activity as the critical ingredient for introducing new products and processes is more crucial for entrepreneurial firms (Stam and Wennberg, 2009). Unlike small business owners, entrepreneurs who strive to grow their firms must invest in innovation, including R&D activities (Samuelsson and Davidsson, 2009). R&D investment can help entrepreneurial firms strengthen core competencies and exploit growth opportunities (Lengnick-Hall, 1992). Successful R&D activities enable entrepreneurial firms to accumulate patents and thus substantially increase the funding level for early financing rounds (Hoenen et al., 2014). Consequently, many scholars are on the advocacy side of R&D activities as they facilitate entrepreneurial performance. At the same time, investing in R&D is a high-risk and high-gain strategy for entrepreneurs. As VCs use the term “burn rate” to describe the high level of R&D intensity, R&D activities burn out the limited cash and resources that are insufficient for entrepreneurial firms in the first place. Thus, deciding how much to invest in R&D is a crucial challenge for entrepreneurs.

For entrepreneurial firms, the case of overinvestment in R&D may be even more severe than in stable and mature firms. Without agency conflict problems, entrepreneurs spend more on R&D activities, driven by entrepreneurial motivation. The intrinsic motivation and long-term approach encourage the founder-CEOs to pursue the optimal shareholder-value maximizing strategy, such as investing more in R&D and having higher capital expenditures (Fahlenbrach, 2009; Ma et al., 2022). Another reason for the entrepreneurs’ willingness to invest more in R&D is an overestimation of the commercial potential of new technology (Cassar, 2010; Li et al., 2021). This overestimation can be driven by an optimism bias often intrinsic to entrepreneurs valuing their prospects or a strategical incentive to provide a more positive picture to potential investors.

### Institutional investors and venture capital

Extant research shows that institutional investors are more rational and professional than individual investors. Compared with individual investors, institutional investors are less likely to be overconfident traders (Chuang and Susmel, 2011), net buyers of attention-grabbing stocks (Barber and Odean, 2008), or become unduly optimistic in IPO auctions after receiving good returns (Chiang et al., 2011). Unlike institutional investors, individual investors do not incorporate available earnings information in their trades rather than respond to the trailing stock returns presented in automated media articles (Blankespoor et al., 2019) and have more difficulty interpreting conflicting information during crises (Priem, 2021). By contrast, institutional investors are more professional in evaluating accounting information and are critical price setters in capital markets (Nofsinger and Sias, 1999; Griffin et al., 2003; O’Connell and Teo, 2009). Together, the institutional investment portfolios may represent the responses of more rational investors in the market.

Institutional investors can be further split into VCs and non-VCs as they have different investment strategies and expertise. First, venture capital investing is characterized by high variability in the outcomes of new ventures. VCs are “patient” capital, investing in firms having no immediate revenue but the potential for scale (Puri and Zarutskie, 2012). However, other non-VC institutional investors prefer to invest in stably profitable firms. For example, Larkin et al. (2017) find that institutional investors, especially mutual funds, are more likely to hold dividend-smoothing stocks. Second, VCs differ from non-VCs in expertise. A large body of research finds that, by providing their financial, strategic, and management expertise, VCs have value-added effects on investees (Chemmanur et al., 2011; Croce et al., 2013; Jin et al., 2020; Zhang et al., 2020; Han, 2021); however, other non-VC institutional investors focus more on influencing or monitoring management (Johnson et al., 2010). Therefore, VCs may have an advantage in evaluating the businesses in the long run, especially on the strategic side, including R&D decisions.

## Hypothesis development

### The effect of R&D intensity on future institutional investment

Extant literature shows that R&D investment decisions are especially hard for entrepreneurs. On the one hand, R&D is crucial for firm success and survival; on the other hand, R&D may exhaust firms’ resources and speed up their failure (Samuelsson and Davidsson, 2009; Delmar et al., 2013; Ugur et al., 2016). While the prevailing view of R&D efforts is that firms tend to underinvest in R&D, entrepreneurs are more likely to overinvest in R&D due to mitigation in agency conflicts and over-optimism (Fahlenbrach, 2009; Cassar, 2010; Ahuja and Novelli, 2017). All combined, in line with prior research regarding heterogeneity in R&D intensities (Coad, 2019), we expect that both R&D under-and over-investments exist in entrepreneurs.

We argue that an *optimum* level of R&D investments exists concerning shareholders’ value. While marginal costs are constant, there are diminishing marginal returns from R&D investments (Faff et al., 2013). Thus, when marginal benefits cover marginal costs, R&D investments can increase shareholders’ value by exploiting growth opportunities or gaining competitive advantages. Driven by past failure to meet aspirations or prospects, an increase in R&D can signal managers’ efforts to improve financial performance (Chen, 2008). Also, an increase in R&D can accumulate more intellectual properties so that the entrepreneurs may gain competitive advantages (Somaya, 2012). However, when marginal costs outweigh marginal benefits, an increase in R&D investments undermines shareholders’ value. Theoretically, managers should decide on an *optimum* level of R&D investments to maximize shareholders’ value.

Investors may hold different attitudes towards the impact of an increase in R&D on shareholders’ value, and we employ institutional investment as the rational response of the capital markets. Institutional investors may evaluate the R&D investments more independently and rationally, free from entrepreneurs’ over-optimism. Also, institutional investors are considered more sophisticated and better-informed traders in the capital market (Schnatterly et al., 2008; Cai et al., 2010; Chiang et al., 2010). Thus, within the *optimum* level of the R&D intensity, an increase in R&D investments increase shareholders’ value, and thus future institutional investments should increase. However, as the R&D intensity exceeds the *optimum* level, the overinvestment in R&D deteriorates shareholders’ value, and correspondingly, future institutional investments should begin to decrease. Thus, we expect the relationship between R&D intensities and future institutional investments to be inverted-U, which leads to our Hypothesis 1:

*Hypothesis 1:* There is an inverted-U relationship between R&D intensity and future institutional investment.

However, the effects on future institutional investments will likely vary depending on institutions’ preferences and expertise. To better understand the inverted-U shape relationship between R&D intensity and future institutional investment, we further split institutions into VCs and non-VCs. The *optimum* level of R&D intensity should vary between VC and non-VC for the following reasons.

First, VCs prefer longer-term benefits from entrepreneurs’ success in scaling. Prior research documents a positive return on R&D investments both in the short-term and long-term (Chambers et al., 2002; Eberhart et al., 2004). VCs would pursue long-term benefits of R&D investments rather than short-term excess positive stock because they are more “patient” investors willing to appreciate massive returns after investees manage to scale. By contrast, facing higher short-term performance pressure, non-VCs may be more sensitive to increased earnings and return volatility associated with R&D investments (Amir et al., 2007; Gharbi et al., 2014). For example, as a kind of non-VCs, transient investors maximize short-term profits by actively trading to extract excess stock returns. Therefore, the activity that entrepreneurs increase R&D intensity to generate long-term value at the expense of short-term profitability would be better admitted by VCs.

Second, VCs’ expertise can contribute to mitigating information asymmetry and commercializing innovation. VCs’ portfolios are much more concentrated than non-VCs’ (Chan and Park, 2013; Fulkerson and Riley, 2019); VCs’ specialization strategy may be represented by developing specialized expertise (Dimov and De Clercq, 2006). Their specialized expertise allows for a better understanding of the complexities associated with industries (Dimov and De Clercq, 2006). It thus may facilitate learning and assessing entrepreneurial firms’ R&D investments in an industry context. Furthermore, VCs’ expertise can contribute to commercializing innovation in many ways. For example, VCs can facilitate professionalization (Hellmann and Puri, 2002), improve total factor productivity (Chemmanur et al., 2011), and provide access to foreign customers (Park and LiPuma, 2020).

Therefore, the *optimum* level of R&D intensity for VCs should be higher because (1) VCs pursue longer-term benefits of R&D investments, and (2) VCs’ expertise help mitigate information asymmetry and commercialize innovation, which leads to our Hypothesis 2:

*Hypothesis 2:* The *optimum* level of R&D intensity is higher for VCs than non-VCs.

### The effect of R&D capitalization on future institutional investments

Research and development capitalization conveys an incremental signal of R&D success. To capitalize, R&D costs have to satisfy all the criteria that evaluate the technical and commercial feasibility. R&D capitalization should decrease information asymmetries regarding the future success of R&D investments. Shortridge (2004) also indicates that the market values successful R&D but not unsuccessful R&D, so we expect institutional investors to value *credible* R&D capitalization positively. However, as summarized earlier, driven by motives to manipulate earnings or convey fake information, R&D capitalization can exceed the creditable level and become *suspicious*. In line with prior research (Dinh et al., 2016; Kreß et al., 2019), we posit that the capital market worries about *suspicious* R&D capitalization, negatively affecting future institutional investments.

Therefore, institutional investors may only positively value the *credible* R&D capitalization but neutrally or negatively value *suspicious* R&D capitalization. In other words, within the *credible* limit, the future institutional investment should increase with the level of R&D capitalization, but decrease as the level of R&D capitalization exceeds the *credible* limit. Thus, we expect the relationship between R&D capitalization and future institutional investments to be inverted-U, which leads to our Hypothesis 3:

*Hypothesis 3:* There is an inverted-U relationship between R&D capitalization and future institutional investment.

As discussed above, *suspicious* R&D capitalization concerns the capital market as it is challenging to grasp R&D projects’ features and predict future performances. Still, VCs should be better at evaluating the true value of *suspicious* R&D capitalization and thus have a higher credit limit of R&D capitalization.

First, VCs’ advantage in knowledge can facilitate the evaluation of the technical and commercial value of capitalized R&D projects. Patent value, for example, requires substantial industry and marketing knowledge (Sapsalis et al., 2006; Fischer and Leidinger, 2014; Hoenen et al., 2014). VCs, especially corporate venture capital (CVC), are more likely to have a richer and more fit industry knowledge (Chemmanur et al., 2014) and thus evaluate the patent value more accurately.

Second, evaluating capitalized R&D requires more time and effort. Given that R&D capitalization can be opportunistic, investors may have to conduct additional financial analysis or consult peer firms. Such research is more practical for VCs than non-VCs because their adoption of specification strategy leads to enhanced monitoring of each investee.

Combined, concerning R&D capitalization, VCs’ expertise, which comes from richer industry knowledge and closer monitoring, helps identify whether *suspicious* R&D capitalization is opportunistic or reflects unexpected R&D success. Thus, the *credible* limit of VCs should be higher than that of non-VCs, which leads to our Hypothesis 4:

*Hypothesis 4:* The *credible* limit of R&D capitalization is higher for VCs than non-VCs.

## Data and research design

### Sample selection

Our sample consists of firms listed on the ChiNext exchange, which aims at promoting the allocation of social funds to innovative businesses and emerging industries. ^2^ The Chinese name “Chuangye Ban” can be directly translated into “Entrepreneur Exchange.” Compared with those listed on the main board, firms listed in ChiNext have a shorter firm age, a higher R&D intensity, and a higher possibility of attracting new funds. ^3^These firms meet the definition of entrepreneurial firms and provide detailed financial data, providing an ideal situation for our research (Hu et al., 2015). We focus on investment decisions in the post-IPO period due to data availability. Iliev and Lowry (2020) show that firms may receive additional financing from VCs in the years after IPO and explain that VC participation after IPO still helps firms exploit value-increasing investments. Thus, both VCs and firms benefit from the value created.

Our data is obtained from the China Stock Market Accounting Research (CSMAR) database between 2009 and 2020, for ChiNext was launched in October 2009. Since R&D cost is our key independent variable, we update and cross-reference the R&D costs data with WIND. All of our continuous variables are winsorized at 1 and 99%. Our initial dataset contains 5,720 observations. We exclude 37 observations of firms in the financial sector. We also drop 208 observations of delisted firms and firms carrying ‘ST’ or ‘*ST’ (i.e., under special treatment or risk alert). Then observations with zero R&D spending are dropped, leading to a further elimination of 97 observations. Lastly, we eliminate 1848 observations for missing sufficient financial or future ownership data. Our final sample comprises 3,530 observations of 700 firms.

### Variables design

#### Institutional ownership

Different types of institutional ownership (*IO*) are the dependent variable in our study. The total institutional ownership (*INST_Ownership*) is measured as the total percentage of institutional shareholding. Next, we classify institutional ownership into VC ownership (*VC_Owneship*) and non-VC ownership (*Non_VC_Ownership*). Our classification of VC follows CSMAR. Specifically, an institutional investor is classified as a VC by CSMAR if (1) its Chinese name includes the meaning of VC, or (2) its scope of business is primarily composed of venture capital investment.

#### R&D investment

R&D intensity (*RDAT*) is measured as the ratio of R&D investment to assets. We do not use R&D normalized by sales because entrepreneurial companies in our sample have low or unstable sales, leading to biased R&D intensity (Paik and Woo, 2017). Similar to Kreß et al. (2019), in each sample year, we define firms that expensed all their R&D as Expensers and firms that capitalize at least part of their R&D as Capitalizers. Next, following Cazavan-Jeny et al. (2011), considering the decision of R&D capitalization, we first split *RDAT* into R&D intensity in Expensers (*RDEX_EXP*) and Capitalizers (*RD_CAP*). For Capitalizers, R&D intensity is further divided into expensed R&D (*RDEX_CAP*) and capitalized R&D (*RDCAP*). Note that expensed R&D for Capitalizers and expensed R&D for Expensers have different natures: expensed R&D for Capitalizers have failed to satisfy capitalization criteria. By contrast, some expensed R&D for Expensers may meet capitalization criteria, but the Expensers still choose to entirely expense R&D to show the market their financial health (Oswald et al., 2021).

#### Control variables

Given that R&D intensity or R&D capitalization is unlikely to be the sole determinant of future institutional investments, we control for a vector of firm-specific characteristics. Guo and Jiang (2013) provide empirical survey evidence, finding that VCs consider entrepreneurial firms’ product, market, and financial aspects when making investment decisions. Other research shows that institutional investment choice is associated with firm size, cash dividend yield, firm age, market risk, state ownership, leverage, and market-to-book ratio (Gompers and Metrick, 2001; Yan, and Zhang, 2009). Thus, we include the ratio of the book value of debts to assets (*LEV*), the annual growth rate of revenue (*SALES_GR*), largest shareholder’s ownership (*Top1*), state ownership (*SO*), the natural log of the market value of equity at fiscal year-end (*SIZE*), market-to-book ratio (*MB*), the ratio of net income to average book value of assets (*ROA*), market risk calculated from a market model (*BETA*), the cash dividend divided by share price (*DP*), excess annual return (*EXCESSR*), firm age measured by the number of months since establishment (*AGE*). We also include industry and year variables to control industry and time effects. *INDUSTRY* is a dummy variable for industry sectors. Note that *INDUSTRY* is classified using China Securities Regulatory Commission (CSRC) Industry Codes, which classify firms into 18 categories. Definitions of all variables are in Appendix1.

### Models

To test Hypothesis 2, we run the following OLS regression to examine the relation between R&D intensity and future institutional investment:


$$\begin{array}{l} IO_{\mathrm{it}+1}=\beta_{0}+\beta_{1}RDAT_{\mathrm{it}}+\left(\beta_{2}\mathrm{RDAT}\_\mathrm{Squar}e_{\mathrm{it}}\right)\\ +\theta_{n}\mathrm{Control}s_{\mathrm{it}}+\mathrm{YEAR}+\mathrm{INDUSTRY}+\varepsilon \end{array}$$

where, *i* denotes firm *i* and *t* denotes year *t*. *IO_it + 1_* is the 1-year-forward institutional ownership, which could be *INST_Ownership _it + 1_*, *VC_Ownership _it + 1,_* or *Non_VC_Ownership_it + 1_*. *RDAT_it_* is R&D intensity measured as R&D divided by assets, and *RDAT_Square_it_* is *RDAT_it_* squared. *Controls_it_* is a vector of control variables as discussed above. *YEAR* and *INDUSTRY* are included to control year and industry fixed effects, respectively, and then estimate clustered standard error at the firm level. If the relationship between R&D intensity and future institutional investment is inverted-U, we expect the coefficient on *RDAT_it_* to be significantly positive and *RDAT_Square_it_* to be significantly negative.

We measure the *IO* for year *t +* 1 for the following reasons. First, institutional investors need time to devote effort to ex-ante project selection. Survey evidence by Guo and Jiang (2013) finds that for over 85% of the interviewed VCs in China, assessing projects before making investment decisions takes more than 3 months. Second, the 1-year-forward institutional investment is not much affected by the 1-year-forward R&D investment because the 1-year-forward institutional investment is disclosed on December 31st, earlier than the announcement date of the 1-year-forward annual reports. Our approach is similar to Bushee and Noe (2000) and Harjoto et al. (2017).

To test Hypothesis 3, 4, we split R&D intensity as discussed above to examine the relationship between each category of R&D intensity and future institutional investment:


$$\begin{array}{l} IO_{\mathrm{it}+1}=\beta_{0}+\beta_{1}\mathrm{RDCA}P_{\mathrm{it}}+\left(\beta_{1}\mathrm{RDCAP}\_\mathrm{Squar}e_{\mathrm{it}}\right)\\ +\theta_{n}\mathrm{Control}s_{\mathrm{it}}+\mathrm{YEAR}+\mathrm{INDUSTRY}+\varepsilon \end{array}$$

where, *i* denotes firm *i* and t denotes year *t*. *RDCAP_it_* is capitalized R&D divided by assets, and *RDCAP_Square_it_* is *RDCAP_it_* squared. In Model 2, we control *RDEX_EXP_it_* measured by expensed R&D divided by assets for expensers and *RDEX_CAP_it_* measured by expensed R&D divided by assets for capitalizers, and the rest of the control variables in Model 2 are defined the same as those in Model 1. If the relationship between R&D capitalization and future institutional investment is inverted-U, we would expect the coefficient on *RDCAP_it_* to be significantly positive and *RDCAP_Square_it_* to be significantly negative.

## Main results

### Descriptive statistics

Table 1 presents the summary statistics for all the variables. The mean value of *RDAT* is 2.99%, which indicates that the average annual investment in R&D is approximately 2.99% of assets. Similarly, the mean value of capitalized R&D (*RDCAP*) is 0.26%, indicating that firms capitalize approximately 0.26% of average assets from R&D investments. Note that for the capitalizer subsample, the mean value of capitalized R&D (*RDCAP*) is much higher since only approximately 25% of firms choose to capitalize R&D. The mean value of our sample’s firms’ institutional ownership is 4.78%, in which VCs’ is 1.40% and non-VCs’ is 3.39%. The descriptive statistics for the control variables are also reported in Table 1.

**Table 1:** Descriptive statistics of variables.

Variable	*N*	Mean (%)	Min (%)	P25 (%)	P50 (%)	P75 (%)	Max (%)	SD
INST_Ownership	3,530	4.78	0.00%	0.88%	3.17%	6.51%	67.40%	0.0647
VC_Ownership	3,530	1.40	0.00	0.00	0.00	0.89	63.50	0.0512
NON_VC_Ownership	3,530	3.39	0.00	0.00	2.21	5.05	55.00	0.0407
RDAT	3,530	2.99	0.26	1.61	2.41	3.70	22.67	0.021
RDCAP	3,530	0.26	0.00	0.00	0.00	0.14	13.07	0.00639
LEV	3,530	30.40	3.49	16.60	28.10	42.60	73.50	0.168
SAL_GR	3,530	17.90	−199.50	2.22	16.60	31.00	288.50	0.303
TOP1	3,530	30.20	8.09	21.00	28.50	38.60	62.50	0.124
SO	3,530	0.0526	0	0	0	0	1	0.223
SIZE	3,530	22.23	20.74	21.67	22.15	22.7	24.7	0.778
MB	3,530	2.358	1.04	1.492	1.954	2.782	8.319	1.307
ROA	3,530	4.07	−35.20	2.21	4.64	7.25	18.60	0.0721
BETA	3,530	1.293	0.518	1.099	1.281	1.479	2.185	0.312
DP	3,530	0.57	0.00	0.13	0.37	0.78	3.55	0.00662
EXCESSR	3,530	−0.25	−73.60	−24.10	−8.08	13.10	202.70	0.418
AGE	3,530	1.851	0.27	1.45	1.82	2.2	4.16	0.58
RDEX_CAP	3,530	0.81	0.00	0.00	0.00	1.09	7.37	0.0157
RDEX_EXP	3,530	1.88	0.00	0.00	1.62	2.73	10.70	0.0199

Table 2 provides pairwise correlations for the main variables. As a preliminary result, we show the co-variation between our main variables: for the dependent variables, the correlation coefficient of *RDAT* with VC ownership (*VC_Owneship*) is significantly positive (0.032, value of *p* <0.05), while insignificant with institutional ownership (*INST_Ownership*) or non-VC ownership (*Non_VC_Ownership*). This insignificant finding is consistent with our hypothesis that R&D intensity and institutional ownership may not be linearly positive or negative. We also show that the co-variation between RDCAP and all three dependent variables is significantly positive (0.059, 0.029, and 0.064, value of *p* <0.01, < 0.10, and < 0.01), which suggests that compared with R&D intensity, capitalized R&D may have a stronger effect on future institutional investments. We calculate the variance inflation factors (VIF), and VIF range from 1 and 1.50, much less than 10, mitigating multicollinearity concerns.

**Table 2:** Correlation analysis.

Variable								
INST_Ownership	**(1)**	1						
VC_Ownership	**(2)**	0.689^***^	1					
NON_VC_Ownership	**(3)**	0.688^***^	−0.027^*^	1				
RDAT	**(4)**	0.008	0.032^**^	−0.0200	1			
RDCAP	**(5)**	0.059^***^	0.029^*^	0.064^***^	0.424^***^	1		
RDEX_CAP	**(6)**	0.027^*^	0.015	0.0250	0.507^***^	0.610^***^	1	
RDEX_EXP	**(7)**	−0.032^**^	0.0110	−0.060^***^	0.465^***^	−0.390^***^	−0.484^***^	1

This table reports the correlation coefficients on the main variables defined previously. The triangle presents the Pearson correlation efficients. ^*^, ^**^, and ^***^ indicate significance at the 0.10, 0.05, and 0.01 level (two-tailed), respectively.

### Regression results

Table 3 reports estimates concerning Model 1. We report linear and non-linear regression results for each type of institutional ownership. When we restrict the relationship to be linear, our result in column (1) shows that the effect of R&D intensity on future institutional ownership is significantly positive (0.1880, value of *p* <0.01), suggesting that at the aggregate level, R&D intensity increases shareholders’ value by mitigating R&D underinvestment. Next, in column (2), our quadratic result shows the significant negative coefficient on squared R&D Intensity (*RDAT_Square*; −1.4822, value of *p* < 0.01), indicating an inverted-U relationship between R&D intensity and future institutional ownership. These results support Hypothesis 1, an inverted-U relationship exists between R&D intensity and future institutional ownership.

**Table 3:** Future institutional investment and R&D intensity.

	Ownership_it + 1_					
	(INST)		(VC)		(NON-VC)	
	**(1)**	**(2)**	**(3)**	**(4)**	**(5)**	**(6)**
**RDAT**_**it**_	**0.1880** ^***^	**0.3575** ^***^	**0.1408** ^***^	**0.1971** ^***^	**0.0352**	**0.1551** ^***^
	(0.052)	(0.086)	(0.038)	(0.054)	(0.031)	(0.060)
**RDAT_Square**_**it**_		**−1.4822** ^***^		**−0.4928**		**−1.0490** ^***^
		(0.548)		(0.312)		(0.390)
LEV_it_	0.0416^***^	0.0417^***^	0.0244^***^	0.0244^***^	0.0168^***^	0.0169^***^
	(0.007)	(0.007)	(0.005)	(0.005)	(0.005)	(0.005)
SAL_GR_it_	0.0000	0.0001	−0.0024	−0.0024	0.0027	0.0028
	(0.003)	(0.003)	(0.002)	(0.002)	(0.002)	(0.002)
TOP1_it_	−0.0070	−0.0076	−0.0000	−0.0002	−0.0080	−0.0084^*^
	(0.007)	(0.007)	(0.005)	(0.005)	(0.005)	(0.005)
SO_it_	0.0255^***^	0.0253^***^	0.0235^***^	0.0235^***^	−0.0006	−0.0007
	(0.006)	(0.006)	(0.006)	(0.006)	(0.002)	(0.002)
SIZE_it_	0.0127^***^	0.0128^***^	0.0019^*^	0.0019^**^	0.0104^***^	0.0104^***^
	(0.002)	(0.002)	(0.001)	(0.001)	(0.001)	(0.001)
MB_it_	−0.0012	−0.0013	−0.0001	−0.0001	−0.0008	−0.0009
	(0.001)	(0.001)	(0.001)	(0.001)	(0.001)	(0.001)
ROA_it_	0.0073	0.0071	−0.0153^*^	−0.0153^*^	0.0243^***^	0.0241^***^
	(0.013)	(0.013)	(0.008)	(0.008)	(0.009)	(0.009)
BETA_it_	−0.0067^*^	−0.0069^**^	0.0048^**^	0.0048^**^	−0.0115^***^	−0.0116^***^
	(0.003)	(0.003)	(0.002)	(0.002)	(0.003)	(0.003)
DP_it_	0.1205	0.1195	0.1116	0.1113	0.0042	0.0035
	(0.156)	(0.156)	(0.106)	(0.106)	(0.108)	(0.108)
EXCESSR_it_	0.0039	0.0038	−0.0001	−0.0001	0.0038^**^	0.0037^**^
	(0.003)	(0.003)	(0.002)	(0.002)	(0.002)	(0.002)
AGE_it_	0.0055^**^	0.0056^**^	0.0036^**^	0.0036^**^	0.0014	0.0014
	(0.002)	(0.002)	(0.002)	(0.002)	(0.001)	(0.001)
INTERCEPT	−0.2554^***^	−0.2591^***^	−0.0564^**^	−0.0576^***^	−0.1887^***^	−0.1913^***^
	(0.036)	(0.036)	(0.022)	(0.022)	(0.024)	(0.024)
INDUSTRY.F.E	YES	YES	YES	YES	YES	YES
YEAR.F.E	YES	YES	YES	YES	YES	YES
Observations	3,530	3,530	3,530	3,530	3,530	3,530
R-squared	0.129	0.130	0.093	0.094	0.131	0.132

Standard errors clustered at the firm-level appear in parentheses. ^***^, ^**^, and ^*^ indicate significance at the 1, 5, and 10% levels, respectively. Definitions of all variables are provided in Appendix 1.

Similarly, in columns (3–6) of Table 3, we present the linear and non-linear estimates for VC ownership and non-VC ownership. We find an inverted-U relationship between R&D intensity and future non-VC ownership, but a linear relationship exists between R&D intensity and future VC ownership. We argue that the linear relationship suggests that the *optimum* level of R&D intensity for VCs is beyond the distribution of our sample. In other words, the general R&D investment level in the entrepreneurial firms in our sample is still too low for VCs, supporting Hypothesis 2 that the *optimum* level of R&D intensity is higher for VCs than non-VCs.

In Table 4, we report estimates concerning Model 2. Results in columns (1) and (2) show that the coefficient on capitalized R&D (*RDCAP*) is significantly positive (0.4102, value of *p* < 0.01), suggesting that at the aggregate level, an increase in capitalized R&D is positively valued by institutions. In addition, the coefficient on squared capitalized R&D (*RDCAP_Square*) is significantly negative (−5.1882, value of *p* < 0.1), suggesting that the relationship between capitalized R&D and future institutional ownership is also inverted-U. Consistent with Hypothesis 3, these findings support that the institutional investors will only positively respond to R&D capitalization within the *credible* limit.

**Table 4:** Future institutional investment and R&D capitalization.

	Ownership _it + 1_					
	(INST)		(VC)		(NON-VC)	
	**(1)**	**(2)**	**(3)**	**(4)**	**(5)**	**(6)**
**RDCAP**_**it**_	**0.4102** ^***^	**0.6793** ^***^	**0.3029** ^***^	**0.4106** ^***^	**0.1397**	**0.3075** ^**^
	(0.152)	(0.206)	(0.101)	(0.148)	(0.112)	(0.127)
**RDCAP_Square**_**it**_		**−5.1882** ^*^		**−2.0763**		**−3.2355** ^***^
		(2.842)		(2.066)		(1.214)
LEV_it_	0.0320^***^	0.0320^***^	0.0218^***^	0.0219^***^	0.0100^**^	0.0101^**^
	(0.006)	(0.006)	(0.004)	(0.004)	(0.004)	(0.004)
SAL_GR_it_	−0.0002	−0.0001	−0.0023	−0.0023	0.0020	0.0021
	(0.003)	(0.003)	(0.001)	(0.001)	(0.002)	(0.002)
TOP1_it_	−0.0112^*^	−0.0111	−0.0003	−0.0003	−0.0113^***^	−0.0113^***^
	(0.007)	(0.007)	(0.005)	(0.005)	(0.004)	(0.004)
SO_it_	0.0221^***^	0.0221^***^	0.0212^***^	0.0212^***^	−0.0006	−0.0005
	(0.005)	(0.005)	(0.004)	(0.004)	(0.002)	(0.002)
SIZE_it_	0.0153^***^	0.0153^***^	0.0007	0.0007	0.0142^***^	0.0142^***^
	(0.001)	(0.001)	(0.001)	(0.001)	(0.001)	(0.001)
MB_it_	0.0004	0.0004	−0.0002	−0.0002	0.0008	0.0008
	(0.001)	(0.001)	(0.000)	(0.000)	(0.001)	(0.001)
ROA_it_	−0.0179	−0.0184	−0.0034	−0.0036	−0.0134	−0.0137
	(0.012)	(0.012)	(0.007)	(0.007)	(0.009)	(0.009)
BETA_it_	−0.0141^***^	−0.0140^***^	0.0036^*^	0.0036^*^	−0.0172^***^	−0.0172^***^
	(0.003)	(0.003)	(0.002)	(0.002)	(0.002)	(0.002)
DP_it_	−0.0661	−0.0616	−0.0033	−0.0015	−0.0466	−0.0438
	(0.136)	(0.136)	(0.096)	(0.095)	(0.091)	(0.091)
EXCESSR_it_	0.0040^*^	0.0041^*^	−0.0012	−0.0012	0.0054^***^	0.0054^***^
	(0.002)	(0.002)	(0.001)	(0.001)	(0.002)	(0.002)
AGE_it_	0.0060^***^	0.0060^***^	0.0030^*^	0.0030^*^	0.0022^**^	0.0022^**^
	(0.002)	(0.002)	(0.002)	(0.002)	(0.001)	(0.001)
RDEX_CAP_it_	0.0660	0.0468	0.1187^**^	0.1110^*^	−0.0731^*^	−0.0851^*^
	(0.077)	(0.078)	(0.056)	(0.057)	(0.044)	(0.044)
RDEX_EXP_it_	0.1411^**^	0.1524^***^	0.1622^***^	0.1667^***^	−0.0311	−0.0241
	(0.055)	(0.055)	(0.043)	(0.043)	(0.032)	(0.032)
INTERCEPT	−0.3009^***^	−0.3016^***^	−0.0261	−0.0264	−0.2666^***^	−0.2671^***^
	(0.031)	(0.031)	(0.020)	(0.020)	(0.021)	(0.021)
INDUSTRY.F.E	YES	YES	YES	YES	YES	YES
YEAR.F.E	YES	YES	YES	YES	YES	YES
Observations	3,530	3,530	3,530	3,530	3,530	3,530
R-squared	0.131	0.131	0.094	0.094	0.132	0.133

Standard errors clustered at the firm-level appear in parentheses. ^***^, ^**^, and ^*^ indicate significance at the 1, 5, and 10% levels, respectively. Definitions of all variables are provided in Appendix 1.

In columns (3–6) of Table 4, we report estimates of the effects of capitalized R&D (*RDCAP*) on future VC ownership and non-VC ownership. Similar to the results in Table 3, an inverted-U relationship exists only between capitalized R&D and non-VC ownership. We find no *credible* limit of R&D capitalization for VCs, suggesting that VCs may be better at evaluating R&D capitalization. Findings in Tables 3, 4 combined indicate that VCs are less concerned with the risks and uncertainty in R&D investments, and this is consistent with the literature that the VCs are long-term oriented and have specialized expertise.

## Discussion

Current research has documented both the positive and negative effects of R&D activities. For example, entrepreneurs can use R&D investment to develop innovative products and advance technologies to achieve better financial and market performance (Eberhart et al., 2004; Guo et al., 2006; Artz et al., 2010; Grimpe et al., 2017). Meanwhile, R&D may exhaust firms’ resources and speed up their failures (Samuelsson and Davidsson, 2009; Delmar et al., 2013; Ugur et al., 2016).

Inspired by previous research, we argue that considering shareholders’ value, investors may have an *optimum* level of R&D intensities and a *credible* limit of R&D capitalization, which reflects the R&D success. Our research is designed in several different ways. First, we propose a double-edged perspective, leading to an inverted-U relationship instead of a simple linear relationship. Second, we focus on the investment of institutional investors and VCs, who are more sophisticated in the capital market. Third, we examine both the effects of R&D intensity and R&D capitalization.

Using a large panel dataset for listed entrepreneurial firms in ChiNext, we explore the double-edged sword effect of R&D intensity and capitalization on attracting more institutional investments. Our examination of the first hypothesis confirms that, after R&D intensity exceeds the *optimum* level, institutional investors will recognize it as overinvestment and decrease their future investment. Furthermore, our examination of the second hypothesis shows that VC investors hold a higher *optimum* level of R&D intensity. The testing of the third hypothesis moves on to the double-edged sword effect of R&D capitalization for entrepreneurial firms and finds that institutional investors treat R&D capitalization as *suspicious* after a *credible* limit. Accordingly, the fourth hypothesis shows that VCs have a higher *credible* limit of R&D capitalization for entrepreneurial firms than non-VCs.

## Conclusion

Using the quadratic regression, we show an inverted-U relationship between R&D intensity and future institutional investment and between R&D capitalization and future institutional investment. The inverted-U relationships suggest that an increase in entrepreneurial firms’ R&D intensity and R&D capitalization will positively impact institutional investors’ financial decisions up to a certain level, which we refer to as the *optimum* level or the *credible* limit, after which an increase in entrepreneurial firms’ R&D intensity and R&D capitalization will discourage future institutional investment. Therefore, we conclude that both R&D intensity and R&D capitalization have a double-edged sword effect on institutional investment in entrepreneurial firms.

Another critical aspect of this paper is separating VC investors from non-VC investors. We argue that the *optimum* level for VCs should be higher than non-VCs due to their different investment strategies and expertise. Our results show that after the *optimum* level or the *credible* limit, an increase in R&D intensity and R&D capitalization will depress non-VCs’ willingness to invest, while this converting process does not happen to VCs. Our findings indicate that VCs are less concerned about R&D overinvestment and *suspicious* R&D capitalization due to their information advantage.

### Implication

These empirical findings provide novel insights into R&D intensity and capitalization. Recent times have witnessed substantial growth in innovative efforts and outcomes (Lăzăroiu et al., 2021; Nica and Stehel, 2021; Suler et al., 2021). In contrast to the conventional view that entrepreneurs are encouraged to invest in R&D as much as possible, we show that institutional investors are discouraged by R&D overinvestment and *suspicious* R&D capitalization. Furthermore, VCs are less likely to worry about risks and uncertainty in R&D investments.

This research can be helpful not only to academics but also to practitioners. Entrepreneurial firms should consider investors’ characteristics and preferences when considering new investments. Our results imply that entrepreneurial firms may have to ensure their R&D intensity and capitalization are reasonable in the eyes of potential investors. In the early stage of new ventures, VCs play an essential role in the rounds of financing, under the pressure from existing competitors and other new ventures, entrepreneurs may adopt relatively more aggressive strategies. In later stage with the development and growth, especially after going public, entrepreneurial firms may then consider decreases in R&D as non-VCs hold a lower level of acceptance for R&D intensity and capitalization.

### Limitation and future research

This paper has several limitations, pointing to areas for future research. First, although we document that R&D overinvestment and *suspicious* R&D capitalization concern institutional investors, the possible and plausible mechanisms leading to R&D overinvestment in entrepreneurial firms need further investigation. Many potential arguments are proposed in existing literature, but there is limited empirical research that analytically evaluates the resource allocation process in entrepreneurial firms. Second, we focus on the financial implications of R&D overinvestment and *suspicious* R&D capitalization. Future research could broadly explore implications in line with this work, such as group management, market growth, and competition strategy. Third, our sample contains relatively large publicly traded entrepreneurial firms in China. It is unclear whether our theory would apply in other countries or entrepreneurial firms that fail to go public. Speculatively, trading environments and investors of developing countries differ from those of developed countries. Future research would be helpful to extend our results by giving closer scrutiny to other institutional contexts.

## Data availability statement

Publicly available datasets were analyzed in this study. This data can be obtained from the China Stock Market Accounting Research (CSMAR) database. This data can be found at: https://www.gtarsc.com/.

## Ethics statement

Ethical review and approval were not required for this study on human participants in accordance with the local legislation and institutional requirements.

## Author contributions

YF, CM, and JM contributed to the conception and design of the study. CM performed the statistical analysis of multiple regression under the supervision of YF. All authors contributed to the article and approved the submitted version.

## Conflict of interest

The authors declare that the research was conducted in the absence of any commercial or financial relationships that could be construed as a potential conflict of interest.

## Publisher’s note

All claims expressed in this article are solely those of the authors and do not necessarily represent those of their affiliated organizations, or those of the publisher, the editors and the reviewers. Any product that may be evaluated in this article, or claim that may be made by its manufacturer, is not guaranteed or endorsed by the publisher.

## Footnotes

^1^See: Criteria for capitalization in IAS 38: https://www.ifrs.org/issued-standards/list-of-standards/ias-38-intangible-assets.html/content/dam/ifrs/publications/html-standards/english/2021/issued/ias38/.

^2^See: http://www.szse.cn/English/products/equity/ChiNext/

^3^See a comparison of listing standards: http://www.szse.cn/English/listings/standards/.

## Appendix I

Variables definitions.

**Table:**

Variable	Definition	Measurement
**Dependent variables**		
INST_Ownership	Institutional ownership	Shares owned by institutional investors/total shares
VC_Ownership	VC ownership	Shares owned by VC/total shares
NON_VC_Ownership	NON-VC ownership	Shares owned by non-VC/total shares
**Independent variables**		
RDAT	R&D intensity	R&D costs/assets
RDCAP	Capitalized R&D	Capitalized R&D costs/assets
**Control variables**		
LEV	Asset-liability ratio	Debts/assets
TOP1	Largest shareholder’s ownership	Largest shareholder’s shares /total shares
SO	State ownership	1 if the firm is a state-owned enterprise, 0 otherwise
SAL_GRO	Sales growth	Ln (sales_t_/sales_t-1_)
SIZE	Size of equity	Ln (market value of equity)
MB	Market to book ratio	Market value of equity/book value of equity
ROA	Return on assets	Net profits/(assets_t_ + assets_t + 1_)/2
BETA	Firms’ market risk	Calculated from a market model using daily stock returns of past 180 days
DP	Cash dividend yield	Annual cash dividend yield per share /share price on December 31^th^
EXCESSR	Annual excess return	Compounded annual stock return- Market return
AGE	Firm’s age since its establishment	Number of months since establishment date
RDEX_CAP	Expensed R&D for capitalizers	Capitalizers’ expensed R&D costs/assets
RDEX_EXP	Expensed R&D for expensers	Expensers’ expensed R&D costs/assets
